# Nonlinear Investigation of Fluorene-Benzothiadiazole Copolymers with Multiphoton Absorption and Highlights as Optical Limiters

**DOI:** 10.1021/acsomega.4c11627

**Published:** 2025-04-16

**Authors:** Leandro H. Zucolotto Cocca, João V. P. Valverde, Elisa B. de Brito, Jilian Nei de Freitas, Maria de F Vmarques, Cleber R. Mendonça, Leonardo De Boni

**Affiliations:** † Institute of Physics of São Carlos, University of São Paulo, CP 369, São Carlos-SP 13560-970, Brazil; ‡ Photonics Group, Instituto of Physics, Federal University of Goias, Goiânia-GO 74690-900, Brazil; § Center for Information Technology Renato Archer, (CTI Renato Archer), Rodovia D. Pedro I, Km 143,6, Campinas-SP 13069-901, Brazil; ∥ Instituto de Macromoléculas Professora Eloisa Mano, IMA, Universidade Federal do Rio de Janeiro, IMA-UFRJ, Av. Horacio MAcedo 2030, Rio de Janeiro 21941-598, Brazil

## Abstract

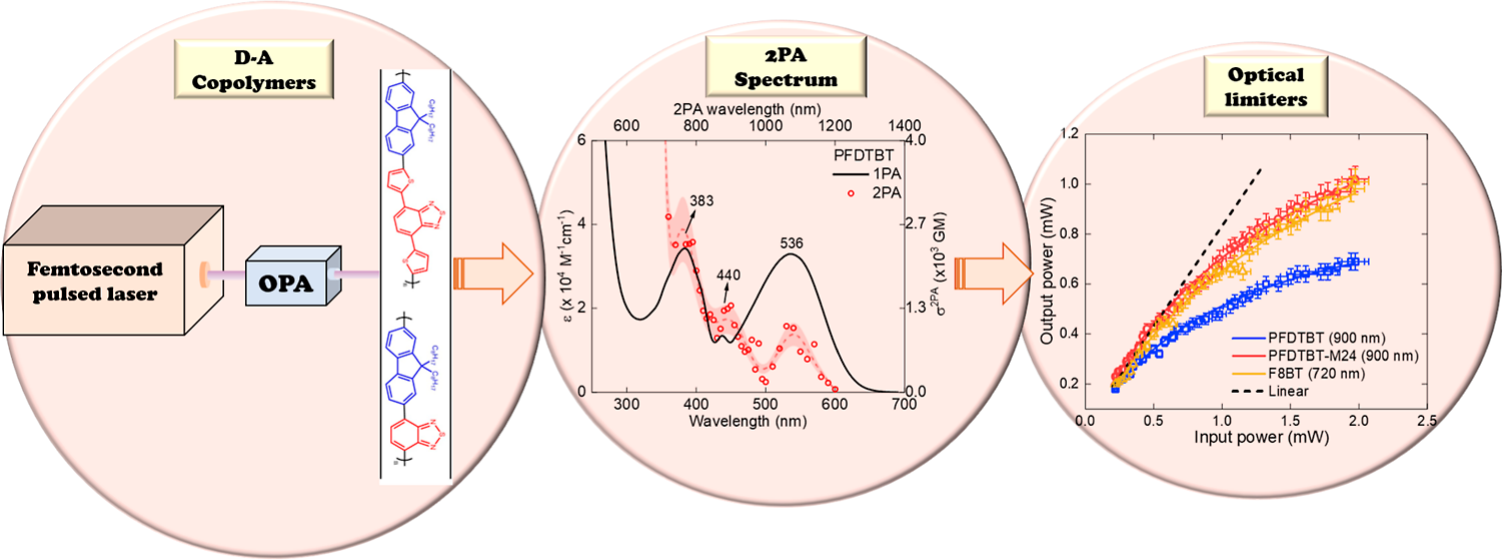

In recent years, conjugated polymers have garnered significant interest due to their versatile optical and electronic properties, including low band gaps and strong absorption in the visible and near-infrared regions. These features, combined with high molar absorptivity and notable photoluminescence and electroluminescence quantum yields, make these materials highly suitable for applications in optoelectronics, nonlinear optics, and other advanced photonic technologies. This study investigates the linear and nonlinear optical properties of three fluorene-benzothiadiazole-based copolymers—**PFDTBT**, **PFDTBT-M24**, and **F8BT**—differentiated by their electron-accepting units and polymer chain lengths. Through comprehensive spectroscopic analysis, including one-photon absorption, fluorescence emission, and multiphoton absorption studies, as well as quantum chemical calculations, the research provides insights into how molecular design can be optimized for nonlinear optical performance. The results reveal significant two-photon absorption cross sections and demonstrate the potential of these materials for multiphoton-excited fluorescence and optical limiting applications.

## 1 Introduction

In recent years, donor–acceptor (D–A) conjugated polymers have gained significant attention due to their versatile optical and electronic properties and for present low band gap and absorption in visible and near-infrared regions.^1−3^ Moreover, these materials can present high molar absorptivity^4^ and considerable photoluminescence and electroluminescence quantum yields.^5^ These features make them suitable for various applications,^6^ including optoelectronics,^7^ and nonlinear optics.^8−10^ In addition, materials that may present high nonlinear optical properties can be employed in several areas of science and technology, for example, in the construction of three-dimensional dispositives,^11^ microdevices,^12^ multiphoton excited fluorescence,^13−16^ phototherapy,^17−20^ and optical power limiting.^21,22^

Among these materials, polyfluorene-benzothiadiazole -based copolymers stand out due to their tunable absorption, emission properties, and ability to efficiently transport charges.^23−26^ These features arise from the unique push–pull interaction between electron-donating (D) and electron-accepting (A) units along the polymer backbone, leading to enhanced internal charge transfer (ICT) and extended π-conjugation.^10^ Such characteristics make these materials particularly attractive for exploring advanced photonic applications, including multiphoton absorption^10,27−30^ and optical limiting,^22^ where controlling light–matter interactions is critical.

Regarding nonlinear optics the class of cited copolymers stands out owing to the large delocalized π-electron systems, this character can lead to high values of third-order nonlinear optical parameters.^22^ Specifically, regarding two-photon absorption it is known that some parameters can influence the two-photon absorption cross-section, examples of this are effective numbers of π-electrons, transition and permanent electric dipole moments^31−33^ the conjugation length and charge separation.^34^

In this scenario, this study aims to investigate the linear and nonlinear optical properties of three well-known polyfluorene-thiophene-benzothiadiazole and polyfluorene-benzothiadiazole, respectively-based copolymers—**PFDTBT**, **PFDTBT-M24**, and **F8BT**—which are distinguished by their differing electron-accepting units and polymer chain lengths. While **PFDTBT** and **PFDTBT-M24** incorporate the 4,7-dithiophen-2-yl-benzo[1,2,5]thiadiazole (**TBT**) as the acceptor, **F8BT** features benzo[1,2,5]thiadiazole (**BT**). These structural differences directly influence their optical responses, offering insights into how molecular design can be tailored to optimize nonlinear optical performance. Considering the optical properties of these materials and their potential impact on optics and photonics, it is crucial to first understand how their chemical structures contribute to enhancing their optical performance for future applications. In this way, the novelty of this work lies in the linear and nonlinear optical characterization of these materials. Specifically, one of the key parameters determined is the two-photon absorption cross-section spectrum. With this information, we can not only gain insights into the influence of chemical features but also guide the development of materials for targeted applications. Another important point to highlight is the potential of these materials as optical limiters. While these materials are well-known and have been used in several scientific areas, to the best of our knowledge, there are no studies reporting their capability to function as optical limiters for ultrafast pulses, thus showing another novelty of the work.

Here, a comprehensive spectroscopic analysis was performed to determine the linear and nonlinear optical processes of these copolymers. This includes one-photon absorption (1PA), fluorescence emission, two-photon absorption (2PA) (employing the well-known femtosecond tunable Z-Scan technique), and multiphoton-excited fluorescence (2PA and 3PA). Additionally, quantum chemical calculations were used to complement experimental findings, providing deeper insight into the electronic transitions and the nature of the observed absorption bands. By correlating structural features with the resulting optical properties, this study aims to further our understanding of the factors driving efficient multiphoton absorption, with potential applications in optical limiting. The results show that the investigated materials present a two-photon absorption cross-section ranging from ∼220 to ∼1300 GM. In addition, this study also demonstrates the ability of the materials to present multiphoton excited fluorescence and the capacity to be employed as optical limiting.

## 2 Materials and Methods

### 2.1 Copolymers

**Figure 1:**
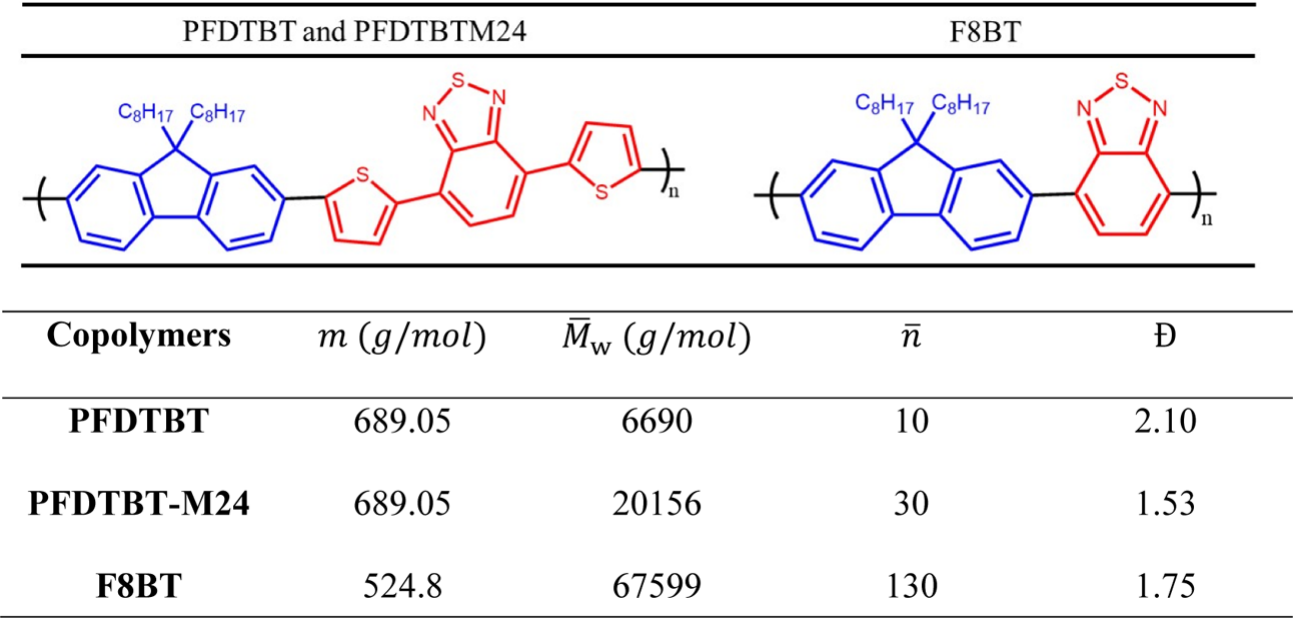
–Representation of chemical structure of D–A polyfluorene-based copolymers. The copolymers have a 9,9-dioctyl-9*H*-fluorene moiety (blue) acting as an electron-donating group, a 4,7-dithiophen-2-yl-benzo[1,2,5]thiadiazole moiety (red) acting as an electron acceptor for **PFDTBT** and **-M24**, and benzo[1,2,5]thiadiazole (red) unit acting as an electron acceptor for **F8BT**.

The three polyfluorene-benzothiadiazole-based copolymers, widely recognized and whose chemical structures are shown in Figure 1, were spectroscopically investigated. Brito et al. provide details of the synthesis of the copolymer poly(9,9-dioctylfluorene-alt-benzothiadiazole) (**F8BT**) [ref (23) The methodology for the synthesis of poly([2,7-(9,9-bis(2-ethylhexyl)-fluorene)]-alt-[5,5-(4,7-di-2′-thienyl-2,1,3-benzothiadiazole)]) (**PFDTBT**) is presented in the Supporting Information, section SI1. To facilitate identification, the nomenclature commonly adopted in the literature was employed for the samples: **PFDTBT**, **PFDTBT-M24**, and **F8BT**. These copolymers exhibit an alternating sequence of 9,9-dioctyl-9*H*-fluorene moiety, functioning as an electron donor (D) and moieties that serve as electron acceptors (A), thereby forming a D–A push–pull structure. The copolymers differ in acceptor units and average chain length; for **PFDTBT** and **PFDTBT-M24**, the acceptor unit is **TBT**, whereas for **F8BT**, it is **BT**. A summary of the chemical structure scheme of the copolymers, including the monomer molar mass (*m*), weight-average molar mass (), average chain length (n̅), and dispersity (*D̵*), is presented in Figure 1.

### 2.2 Spectroscopic Measurements

To determine the linear optical properties of the copolymers, dilute solutions were prepared at a concentration of approximately 10^–4^ mol/L in chloroform. The 1PA spectrum was obtained using a UV–vis spectrophotometer (Shimadzu, UV-1800 model) and a 2.0 mm quartz cuvette. The fluorescence emission spectrum was acquired using a fluorometer (Hitachi, F7000 model) and a 10.0 mm quartz cuvette, with the solution concentration being further diluted (∼10^–6^ mol/L).

Time-resolved fluorescence spectroscopy was used to determine the fluorescence lifetime (τ_fl_) of the samples. A regenerative amplified Yb/KGW femtosecond laser system (Light Conversion, Pharos PH1 model) with a 220 fs temporal width was utilized for sample excitation at 343 nm (third harmonic of the fundamental). The fluorescence emission was monitored perpendicular to the excitation beam as a function of time. The τ_fl_ was obtained using the signal convolution method, which involves the convolution of the instrument response function (IRF) with a monoexponential decay function. For more details on the technique, refer to the following refs (35 and 36).

### 2.3 Two-Photon Absorption Measurements

Copolymer solutions in chloroform were prepared at a concentration of approximately 10^–3^ mol/L to determine their 2PA cross-section (σ_2PA_). No aggregation was observed at this concentration. The solutions were then placed into a quartz cell with a 2.0 mm optical path length and 2PA spectra were obtained using the Z-scan technique in an open-aperture configuration,^37^ where the sample is scanned along the focal plane of a focused Gaussian laser beam. By monitoring the changes in light transmittance, it is observed that linear effects predominate in regions away from the focus, whereas near the focus, where the beam intensity increases, the 2PA process becomes measurable. To eliminate linear contributions, the light transmittance at a specific *z*-position is divided by the transmittance observed when the sample is far from the focal point, thus determining the normalized transmittance (*T*_*N*_). Finally, the 2PA coefficient (β) is determined by fitting the normalized transmittance curve to the following equation.^37^
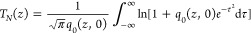
1where , *L* represents the sample thickness, *z*_◦_ is the Rayleigh length, *z* is the sample position, and *I*_◦_ is the pulse intensity. By fitting the experimental curves obtained from the Z-scan measurements using eq 1, it is possible to determine β, which allows the calculation of the σ_2PA_ using the expression , where *N* is the number of molecules per *cm*^3^ and *hν* is the excitation photon energy. σ_2*PA*_ is commonly expressed in Göppert Mayer (GM) units, with .^38^

The σ_2PA_ was measured over a wavelength range of 550 to 1200 nm with a spectral resolution of 10 nm. The measurements were conducted using 150–180 fs laser pulses generated by an optical parametric amplifier (OPA) (Light Conversion, ORPHEUS model). This OPA was pumped by a regenerative amplified Yb/KGW femtosecond laser system (Light Conversion, Pharos PH1 model), delivering 220 fs pulses centered at 1030 nm with a repetition rate of 750 Hz.

### 2.4 Multiphoton-Excited Fluorescence

Multiphoton-excited fluorescence measurements were conducted by exciting the samples at different wavelengths to investigate 2PA and 3PA processes through fluorescence emission. The same laser system and OPA used in the Z-scan technique were employed, operating at a repetition rate of 7.5 kHz. The fluorescence signal was collected perpendicularly to the excitation beam using an optical fiber connected to a spectrophotometer. The laser intensity was adjusted using a rotating polarizer, allowing the analysis of fluorescence emission dependence as a function of excitation beam intensity. The multiphoton absorption mechanism was confirmed by plotting fluorescence intensity (*F*) versus excitation intensity (*I*) on a log–log scale. Since *F*∝*I*^*n*^^39^ where *n* is the photon number involved in the absorption, a linear relationship between the logarithms of *F* and *I* with a slope equal to *n* indicates the simultaneous *n*-photon absorption.

### 2.5 Optical Power Limiter

Two-photon optical limiting measurements were performed using the same experimental setup as in the Z-scan technique, with the sample positioned at the focal plane of the converging lens (*f* = 15 cm). Initially, the laser beam passes through a rotating broadband λ/2 wave plate and a fixed polarizer, allowing precise laser power control. Following, the beam is directed to a beam splitter, where a small fraction of light (∼4%) is used to monitor the input power using a silicon detector. The other portion of the beam (∼96%) is then focused on the nonlinear sample, and a second photodetector monitors the transmitted beam. The entire system was calibrated using a power meter.

### 2.6 Quantum Chemical Calculations

Quantum chemical calculations, employing density functional theory (DFT) and time-dependent density functional theory (TD-DFT) framework, were carried out using the Gaussian 09^40^ package to aid in understanding the experimental results obtained for the copolymers. The investigation was divided into three primary stages: Initially, geometry optimization and vibrational frequency calculations were performed for oligomeric structures (with two and three repeating units) of **PFDTBT** and **F8BT** using the hybrid exchange–correlation functional B3LYP^41^ with the standard basis set 6–311G(d,p).^42^ No imaginary vibrational modes were identified, confirming that the obtained structure corresponds to an actual minimum. It is worth noting that, for the subsequent stages, only the oligomers with two repeating units were used. In the second stage, 1PA properties (oscillator strength and transition energy) were determined considering the 15 lowest-energy singlet electronic transitions. These calculations employed the M06 functional^43^ and the 6–311++G(d,p) basis set. Subsequently, molecular orbitals were determined using the same functional and basis set. All calculations incorporated the effect of the chloroform solvent using the polarizable continuum model (PCM) with the integral equation formalism (IEF) variant.^44,45^ Relevant results regarding the calculations can be found in Section SI2 of the Supporting Information.

## 3 Results and Discussion

**Figure 2:**
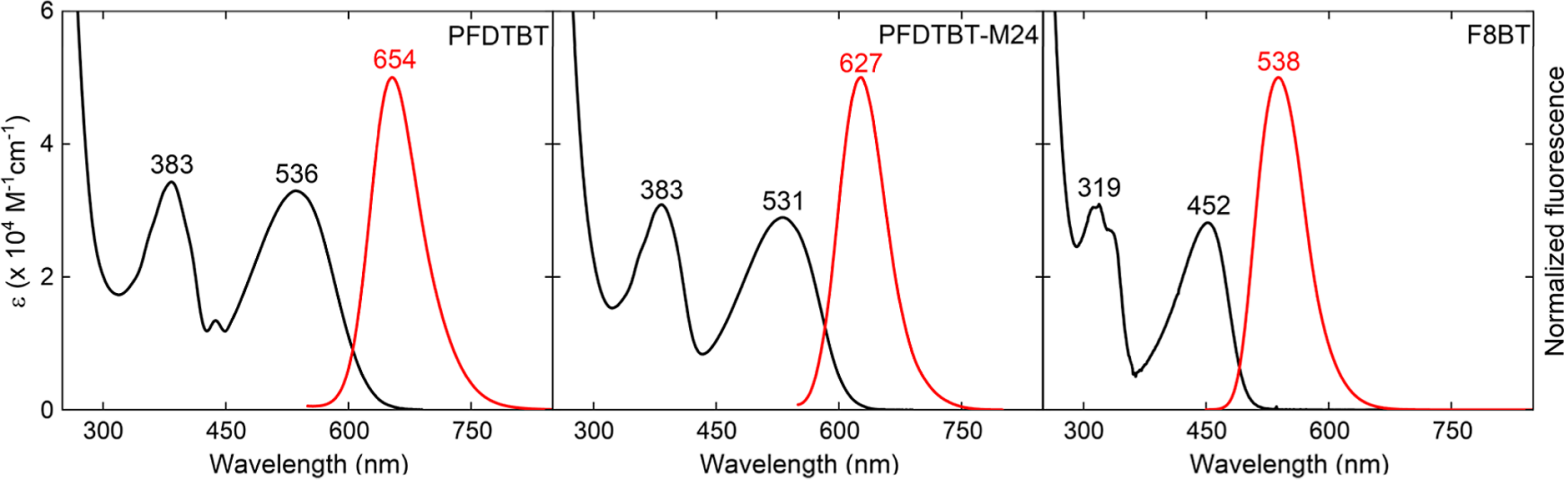
–One-photon absorption (black line: left axis) and normalized fluorescence emission (red lines: right axis) spectra of the copolymers in chloroform.

The 1PA and fluorescence emission spectra are shown in Figure 2. All copolymers exhibited two well-defined “camel-back” type absorption bands in the UV–vis spectral region, with very similar transition intensities, approximately 3.11 × 10^4^ M^–1^ cm^–1^. The lower-energy band is located at ca. 534 nm (2.32 eV) for **PFDTBT** and **M24** and at 452 nm (2.74 eV) for **F8BT**. The higher-energy band is situated at ca. 351 nm (3.53 eV). Both bands are characterized by a charge transfer process, predominantly associated with a π–π* transition.^46^ It was observed that substituting the **BT** moiety with **TBT** in **PFDTBT** and **-M24** samples result in an average bathochromic shift of 0.5 eV compared to **F8BT**. This behavior can be attributed to two key characteristics of the **TBT** moiety: its stronger electron-accepting capability, which favors ICT, and its extended π-conjugated system compared to **BT**.^47^ These properties endow **PFDTBT** and **-M24** with more significant effective conjugation (per monomer) and an increased ICT effect, contributing to the redshift of the bands. Finally, it is worth noting that the absorption spectra obtained in this study are consistent with those reported in other works.^10,46,48^

Based on the results of quantum chemical calculations (see Figure SI1), significant insights into the absorption bands of the copolymers were obtained, revealing that the absorption bands consist of multiple electronic transitions; specifically, the lower-energy band involves two transitions, while the higher-energy band comprises several transitions. Furthermore, a noteworthy aspect of the theoretical results is the presence of certain states with negligible oscillator strengths (0.007 and 0.01 for **PFDTBT** and **F8BT** oligomers, respectively) positioned in the valley of the “camel-back” bands, around 400 nm. The oscillator strength of these transitions is, on average, 25 times weaker than the S_0_ → S_2_ transition. Therefore, based on these values and the observations by Jespersen et al.,^48^ it is suggested that these are transitions weakly accessible by 1PA and significantly impacts the 2PA, as will be shown later.

Figure 2 shows the fluorescence emission spectra (red lines), while Table 1 summarizes key data such as Stokes shift (Δν in eV) and fluorescence lifetime (τ_fl_). The samples exhibited fluorescence emission in the visible spectral region, occurring around 640 nm (red) for **PFDTBT** and **-M24** and 538 nm (green) for **F8BT**. Analyzing the energy difference between the maximum of the lower-energy band and the fluorescence emission peak reveals a significant Δν, with an average value of 0.4 eV. This value suggests a considerable variation in electronic and structural reorganization between the excited state and ground one in each copolymer, as will be discussed later. The τ_fl_ of the samples ranged from 3.2 to 3.9 ns. Considering the experimental uncertainties, there is no statistically significant variation in τ_fl_ among the three copolymers, limiting a more detailed investigation of the excited-state relaxation dynamics. Furthermore, it is essential to compare the lifetimes determined in this study with those reported in the literature for compounds exhibiting multiphoton absorption-induced fluorescence, and potential applications as fluorescent probes and optical limiters. For instance, for fluorescent probes activated by single- or two-photon absorption, reported lifetimes range between 5 and 6 ns.^49^ In the study by Gui-Jiang Zhou, compounds demonstrating a large optical-limiting response exhibited lifetimes between 0.87 and 2.2 ns.^50^ Similarly, Tingchao He et al. reported lifetimes ranging from 1.35 to 2.5 ns for statistical Zn(II)-coordinated copolymers.^51^

**Table 1:** –Linear and Nonlinear Optical Properties of the Copolymers^a^

compounds	Δν̅(eV)	τ (ns)	σ01(GM)/λ01(nm)	σ02(GM)/λ02(nm)	σ03(GM)/λ03(nm)	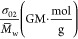
**PFDTBT**	0.42	3.5 ± 0.3	1050/1070	1300/880	2350/770	0.19
**PFDTBT-M24**	0.36	3.9 ± 0.4	130/1060	820/860	900/770	0.04
**F8BT**	0.44	3.2 ± 0.3	26/900	770/730	220/640	0.01

a In the 2PA cross-section section, subscripts 01, 02, and 03 denote the lowest-energy, intermediate (valley), and highest-energy bands, respectively.

**Figure 3:**
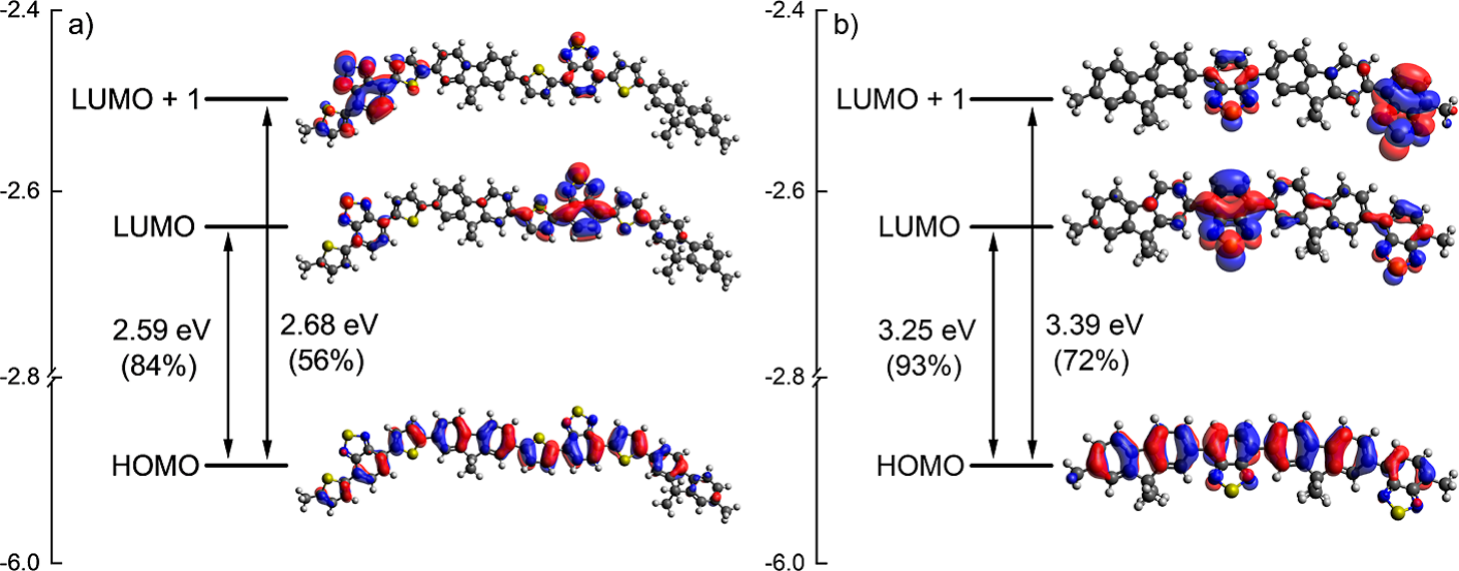
–Molecular orbitals involved in the two lowest-energy electronic transitions, with their respective percentage contributions. Calculations performed at the TD-PCM-M06/6–311++G(d,p) level for **PFBTBT** (a) and **F8BT** (b). The percentages values represent the percentage contributions of HOMO–LUMO and HOMO–LUMO+1 excitations.

Figure 3a,b, respectively, depicts the highest occupied molecular orbitals (HOMO) and lowest unoccupied molecular orbitals (LUMO) of the **PFDTBT and F8BT** oligomers. These orbitals provide the most accurate description (greater percentage contributions) of the two electronic transitions associated with the lowest-energy band. A qualitative analysis of these orbitals reveals that the HOMO of both oligomers exhibits electronic delocalization throughout the entire oligomeric backbone. In contrast, the LUMO and LUMO+1 show charge distribution concentrated in the electron-accepting regions, with a slight difference. In these orbitals (LUMO and LUMO+1), the electronic delocalization is primarily confined to one of the acceptor units. Overall, this behavior suggests that both excitations, HOMO→LUMO and HOMO→LUMO+1, are characterized by an ICT process from the electron-donating moiety (fluorene) to the electron-accepting one (**BT** or **TBT**).^47^

Furthermore, the **PFDTBT** oligomer exhibited a HOMO–LUMO energy gap approximately 1.26 times lower compared to **F8BT**. This was expected, given that this behavior is associated with the more extensive π-conjugated system (per monomer) of the **PFDTBT** and strong electron-accepting group. These findings corroborate with the observed redshifts in the 1PA and with the high Δν values.

**Figure 4:**
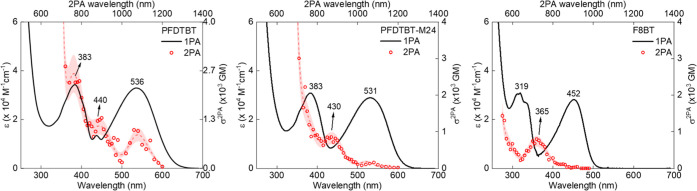
–One-photon absorption (black line) and two-photon absorption (red circles) spectra of the copolymers in chloroform. The dashed red line is a visual guide. The estimated error is approximately 20%.

The 2PA spectra of the copolymers are shown in Figure 4, in meeting with their corresponding 1PA spectra for comparative purposes. Here, the 2PA cross-section (σ_2PA_) is calculated concerning the repeating monomer unit. Experimental results reveal a well-resolved 2PA spectrum, exhibiting substantial σ_2PA_ values, ranging from approximately 220 to 2350 GM for the higher-energy band (see Table 1), covering both the visible and near-infrared spectral regions.

Analysis of the **PFDTBT** sample reveals three distinct 2PA bands and a resonant enhancement effect for wavelengths shorter than 750 nm. Two bands correspond satisfactorily with those observed in the 1PA spectrum, indicating that these states are also allowed via two-photon. A noteworthy feature is the 2PA band centered at approximately 880 nm, corresponds the “camel-back” band valley at 400 nm. This characteristic is also evident in the other two samples, with peaks at 860 nm for **PFDTBT-M24** and 730 for **F8BT**. As indicated by 1PA theoretical results and also observed by Jespersen et al.,^48^ there are indeed electronic states around 400 nm that are weakly accessible via one-photon. However, as suggested by the results, these states are strong allowed by 2PA.

A key term describing the 2PA mechanism is related to the coupling interaction between the first excited state (S_1_) and the *n*-th excited states (S_*n*_, for *n* = 3 and 4). This term, quantified by the two-photon term, is directly proportional to the product of the transition dipole moments coupling S_1_–S_*n*_ and S_0_–S_1_ ().^52,53^ The appearance of these only two-photon accessible excited states can, therefore, be attributed to the strong coupling between S_1_ and the S_*n*_ states (allowed states by two-photon) promoted by the two-photon terms. This phenomenon is commonly observed in noncentrosymmetric copolymers.^10,54−57^

The copolymers **PFDTBT-M24** and **F8BT** exhibit smaller σ_2PA_ compared to **PFDTBT**. When analyzing the intermediate 2PA band (∼800 nm), a decrease of approximately 40% is observed. Also, it is noted for the lower-energy band in both copolymers.

Overall, the results indicate that the presence of the **TBT** moiety in the **PFDTBT** and **-M24** copolymers led to an increase in the σ_2PA_ compared to **F8BT** (which contains the **BT** moiety). This is attributed to the more significant electronic conjugation and electron-accepting capability of the **TBT**, which could explain the smaller 2PA magnitude in **F8BT** relative to **PFDTBT**. However, when comparing the **PFDTBT** and **-M24** copolymers, which show to have identical effective conjugation lengths (per repeat unit), a substantial disparity in σ_2PA_ spectra is observed. This suggests that factors beyond the presence of the **TBT** unit influence 2PA.

One possible explanation for the decreased 2PA observed in the **PFDTBT-M24** and **F8BT** copolymers can be attributed to their longer average polymer chain length (see Table 1). The increased chain length promotes a twisted polymer conformation analogous to a coiling structure. This phenomenon disrupts the coplanarity of the polymer chain, hindering ICT and the cooperative effect between repeater units, which negatively impacts 2PA.^55,56,58^ The tendency of copolymers to form twisted structures due to increased chain length can be observed in Figure SI2. De Boni et al.,^8^ in studies on fluorene-based polymers, support this interpretation. In their work, the authors reported a 38% decrease in the σ_2PA_ for the polymer exhibiting the highest electronic conjugation per monomer, attributing this phenomenon to the loss of structural planarity. Similarly, Vidya and Chetti^55^ and Zou et al.^46^ reported that samples with higher weight-average molar mass exhibit a lower σ_2PA_. Vivas et al.^59^ demonstrated that a conformational change from the coil to the helix in poly(3,6-phenanthrene) reduces approximately 50% in the σ_2PA_, highlighting the impact of structural conformation on 2PA efficiency.

**Figure 5:**
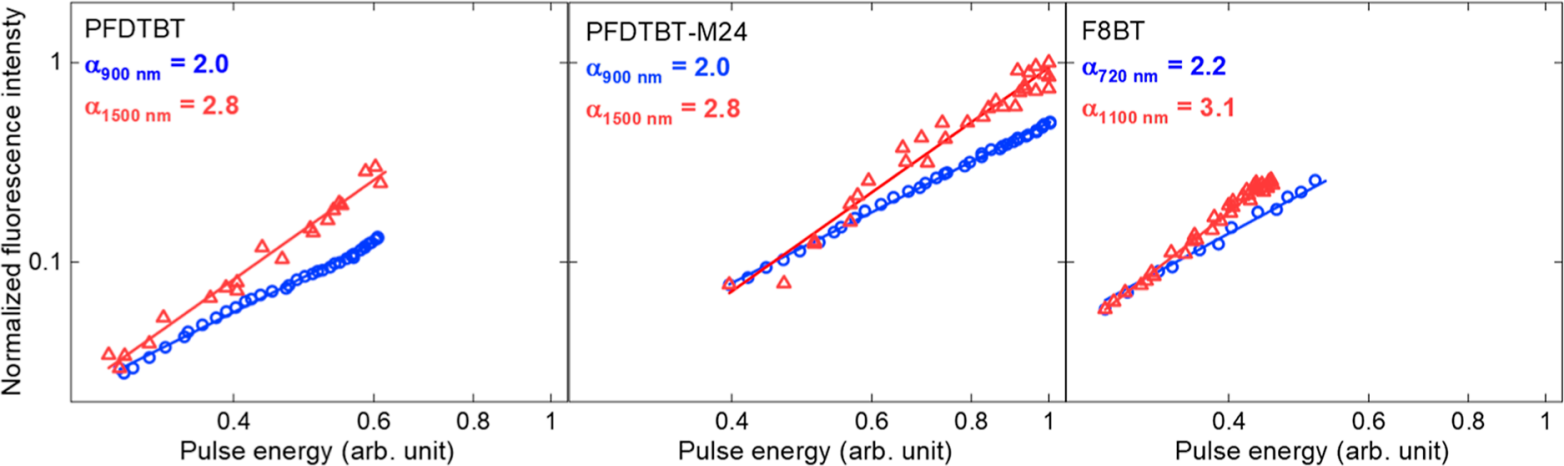
–Fluorescence signal versus laser pulse energy, plotted on a log–log scale, for the copolymers. The excitation wavelengths are 900 and 1500 nm for **PFDTBT** and **-M24**, and 720 and 1100 nm for **F8BT**. The slope (α) of the fitted line indicates the order of the multiphoton absorption process: values close to 2.0 ± 0.2 and 3.0 ± 0.3 correspond to 2PA and 3PA, respectively.

The high σ_2PA_ in the copolymers motivate the investigation of their nonlinear optical properties in multiphoton absorption processes, precisely 2PA and 3PA. This approach is crucial for evaluating the potential of these samples in photonic applications, such as optical limiters. To check this statement, fluorescence emission was collected by exciting the samples at 720, 900, 1100, and 1500 nm, corresponding to 2PA and 3PA. Figure 5 displays the fluorescence intensity curves as a function of incident laser power on a log–log scale. Linear fits of the curves reveal a quadratic (2.0–2.2) and cubic (2.8–3.1) dependence, confirming 2PA and 3PA respectively. These results demonstrate the copolymers’ ability to emit via multiphoton absorption, highlighting their potential as candidates for optical limiter applications.

**Figure 6:**
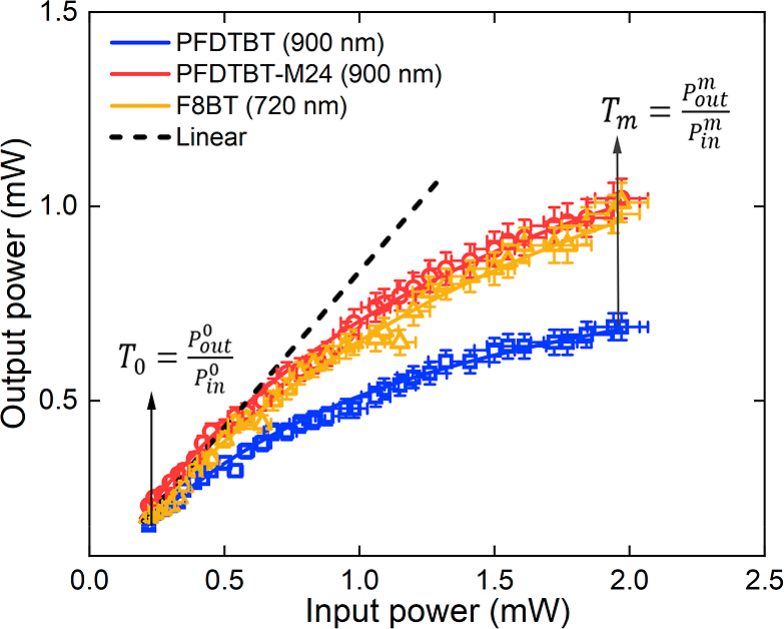
–Optical limiting curves of the copolymers: output power versus input power. The dashed black line represents the ideal case of a material without two-photon absorption, with no optical limiting effect.

Figure 6 presents a typical optical limiting curve for the copolymers, displaying the transmitted (output) laser power as a function of the incident (input) laser power. The optical limiting property of the copolymers was assessed and evaluated in a spectral region characterized by a high σ_2PA_, situated far from any resonant enhancement effects. For the **PFDTBT** and **-M24** samples, measurements were conducted at 900 nm (corresponding to 1380 GM and 770 GM, respectively), whereas for the **F8BT** sample, measurements were carried out at 720 nm (800 GM). The copolymer response exhibits a predominantly linear behavior up to an input power of 0.5 mW. However, for higher input powers (>0.5 mW), the optical limiting effect starts to appear. At this specific power level, the average transmittance of the copolymers is approximately 78%, considering experimental uncertainties. As the input power increases, differences between the samples become more pronounced, with **PFDTBT** demonstrating the most significant reduction in transmittance. For instance, at 1.55 mW, the transmittance of **PFDTBT** drops to about 40%, while for the other copolymers (**PFDTBT-M24** and **F8BT**), transmittance remains around 57%. The optical limiting threshold power for **PFDTBT**, **-M24**, and **F8BT** was determined to be 1.0, 2.2, and 1.9 mW, respectively.

The optical limiting performance was evaluated quantitatively through the figure of merit (),^60^ which is defined as the ratio of the initial transmittance (*T*_0_) to the minimum transmittance (*Tm*) of the sample, with  (*P*_out_^0^ is the initial output power and *P*_in_^0^ is the initial input power) and  (*P*_out_^m^ is the minimal output power and *P*_in_^m^ is the minimal input power). The **PFDTBT** demonstrated the highest FOM value of approximately 2.3, relative to the other copolymers analyzed here (FOM ≈1.8). These results indicate that the FOM is 1.2 to 1.4 times higher than the values commonly observed in different materials in solution.^8,10,61^ Furthermore, significant FOM values (∼2.8–1.8) have been reported for other materials, demonstrating that **PFDTBT** performs at a level consistent with some of the most effective optical limiters found in the literature.^61−63^ Finally, the results demonstrate that the D–A type copolymers investigated in this study exhibit efficient ultrafast optical limiting action via 2PA, making them promising candidates for protecting optical devices against damage caused by high-intensity laser pulses in the studied wavelength range.

## 4 Conclusion

This study investigated linear and nonlinear optical properties of three D–A copolymers polythiophene-based. Using the Z-scan technique, the σ_2PA_ was determined, and the multiphoton absorption capacity of these compounds was assessed through fluorescence excitation measurements. Based on the promising results obtained, the optical limiting performance of the copolymers was evaluated, demonstrating their potential as materials for photonic devices.

Here, it was shown that the investigated copolymers exhibit remarkable σ_2PA_, with values ranging from 220 up to 2350 GM. A strong allowed state, only by 2PA, was identified, located within the “camel-back” band valley (∼870 nm for **PFDTBT**, ∼860 for **-M24** and ∼730 nm for **F8BT**). It was attributed to a strong coupling between the first and nth excited states. Additionally, a strong influence of structural conformation on 2PA was observed. Although the **PFDTBT** and **-M24** copolymers have the same effective electronic conjugation length (per monomer), their different average polymer chain lengths results in a 40% difference in the σ_2PA_. This behavior is attributed to possible conformational twisting that affects the cooperative effect between repeating units. The copolymers also demonstrated the ability for 3PA. This relatively high nonlinear absorption is particularly interesting for photonic devices like optical limiters. Indeed, a reduction of approximately 60% in the input beam transmittance was observed, with a FOM = 2.3. In summary, the results highlight the remarkable properties of D–A copolymers, emphasizing their optical versatility and supporting their potential for applications in nonlinear optics.

## Supporting Information Available

Topic 1: Synthesis process. Topic 2: Quantum chemical calculation details (PDF)
