# Supplementary material for: Nonlinear Investigation of Fluorene-Benzothiadiazole Copolymers with Multiphoton Absorption and Highlights as Optical Limiters
Source: ACS Omega. 2025 Apr 16;10(16):16539–47. doi: 10.1021/acsomega.4c11627 (PMC12044456; doi:10.1021/acsomega.4c11627)
Supplement: Supplementary file 1 — ao4c11627_si_001.pdf [file ao4c11627_si_001.pdf]

Nonlinear investigation of fluorene-benzothiadiazole copolymers with multiphoton absorption and highlights as optical limiters

Leandro H. Zucolotto Cocca[a,b*], João V. P. Valverde[a], Elisa B. de Brito[c], Jilian Nei de Freitas[c], Maria de F. V. Marques[d], Cleber R. Mendonça[a], and Leonardo De Boni[a]

[a] Instituto de Física de São Carlos, Universidade de São Paulo, CP 369, 13560-970 São Carlos, SP, Brazil

[b] Grupo de Fotônica, Instituto de Física, Universidade Federal de Goiás, Goiânia, 74690-900, GO, Brazil

[c] Center for Information Technology Renato Archer, (CTI Renato Archer), Rodovia D. Pedro I, Km 143,6, 13069-901, Campinas, SP, Brazil.

[d] Instituto de Macromoleculas Professora Eloisa Mano, IMA, Universidade Federal do Rio de Janeiro, IMA-UFRJ, Av. Horacio Macedo 2030, RJ, 21941-598, Brazil

*Author to whom correspondence should be addressed: leandro.zucolottococca@gmail.com

SUPPORTING INFORMATION

1. SYNTHESIS PROCESS

Poly([2,7-(9,9-bis-(2-ethylhexyl)-fluorene)]-alt-[5,5-(4,7-di-2′-thienyl-2,1,3-benzothiadiazole)]) (PFDTBT)

1 mmol of 9,9-dioctyl-fluorene-2,7-bis(pinacol ester of diboronic acid) and 1 mmol of 4,7-bis(5-bromothiphen-2-yl)[c][1,2,5]thiadiazole and 0,017 mmol of $Pd(PPh_3)_4$ were added into a round-bottom flask. 12 mL of dry toluene and 8 mL of a 2 M potassium carbonate solution were added to this flask. The mixture was stirred at about 85-90 °C for 48 h under nitrogen protection. After the determined time, the terminating agent (phenylboronic acid) was added to close the chain. The reaction was stirred and heated for another 24 hours. Upon reaching room temperature, the mixture was extracted in chloroform; magnesium sulfate (desiccant agent) was added; the liquid was then filtered, rotary evaporated, and, finally, precipitated in methanol and water in the proportion 10:1. A solid was obtained, which was washed in methanol, water, and methanol. Subsequently, the filtered material underwent Soxhlet extraction in acetone, hexane, and chloroform.

^{1}H-NMR (400 MHz) δ: 8,15 (m, 2H), 7,97 – 7,48 (m, 7H), 7,33 (s, 2H), 2,08 (s, 5H), 1,11 (s, 16H), 0,79 (s, 6H)

Poly{[2,7-(9,9-bis-(2-ethylhexyl)-fluorene)]-alt-[5,5-(4,7-di-2′-thienyl-2,1,3-benzothiadiazole)]} (PFDTBT-M24)

In a round bottom flask, 1 mmol of 9,9-dioctyl-fluorene-2,7-bis(pinacol ester of diboronic acid), 1 mmol of 4,7-bis(5-bromothiphen-2-yl)[c][1,2,5]thiadiazole and 0.017 mmol of $Pd(PPh_3)_4$ were added. To this flask were added 12 mL of dry toluene, 8 mL of a 2 M potassium carbonate solution, and 10 drops of the interfacing agent (Aliquat 336®). The mixture was stirred vigorously at around 85-90 °C for 24 h under nitrogen protection. After the determined time, the terminating agent (phenylboronic acid) was added to close the chain. The reaction was stirred and heated for another 24 hours. Reaching the room temperature, it was extracted in chloroform (liquid-liquid extraction). A desiccant agent (magnesium sulfate - $MgSO_4$) was added to this fraction and left under stirring with a magnetic rod; the liquid was then filtered, rotary evaporated, and, finally, precipitated in methanol and water in the proportion 10:1. A solid was obtained and washed in methanol, water and methanol. The dried sample subsequently underwent Soxhlet extraction in acetone, hexane, and chloroform.

^{1}H-NMR (400 MHz) δ: 8,30 – 7,57 (m, 2H), 7,56 – 7,35 (m, 7H), 2,09 (s, 5H), 1,11 (s, 16H), 0,79 (s, 6H).

2. QUANTUM CHEMICAL CALCULATION DETAILS

Table SI1 presents the optimized structures of the **PFDTBT** and **F8BT** oligomers, obtained through PCM-B3LYP/6-311G(d,p) level calculations, used to determine the molecular orbitals and the one-photon absorption electronic transitions.

Table SI1 - Optimized structures of oligomer **PFDTBT** and **F8BT** obtained through PCM-B3LYP/6-311G(d,p) calculations in chloroform medium.

Oligomer **PFDTBT**				Oligomer **F8BT**			
PCM-B3LYP/6-311G(d,p) Medium chloroform				PCM-B3LYP/6-311G(d,p) Medium chloroform			
C	-4.14754	0.19570	-0.70759	C	8.46201	-1.41266	0.66930
C	-2.90502	-0.69391	-0.66159	C	9.83759	-0.75944	0.53554
C	-1.57481	-0.32083	-0.73159	C	11.08742	-1.31091	0.77704
C	-0.56238	-1.30141	-0.66716	C	12.24603	-0.53960	0.59543
C	-0.93776	-2.65136	-0.52094	C	12.11181	0.79054	0.17326
C	-2.27293	-3.02901	-0.44167	C	10.86163	1.35588	-0.07211
C	-3.26307	-2.04827	-0.51531	C	9.71998	0.57620	0.10940

C	-4.72092	-2.14209	-0.46564	C	8.29814	0.88739	-0.05961
C	-5.57073	-3.24275	-0.33649	C	7.65122	2.05600	-0.46159
C	-6.94473	-3.04042	-0.31666	C	6.26201	2.06899	-0.53429
C	-7.49930	-1.74966	-0.43452	C	5.49605	0.93265	-0.21330
C	-6.62820	-0.64871	-0.56344	C	6.16432	-0.24282	0.18102
C	-5.25839	-0.84587	-0.57500	C	7.54682	-0.25861	0.25939
C	-4.17055	1.19235	0.47213	C	8.31359	-2.61342	-0.29071
C	-4.24565	0.95739	-2.04733	C	8.18861	-1.85981	2.12187
N	4.62074	-3.24847	0.57993	N	3.64626	-1.26115	-1.17926
S	5.66836	-4.36016	1.16891	S	2.33575	-2.14272	-1.60917
N	7.06320	-3.51938	1.00195	N	1.15355	-1.07334	-1.23689
C	6.75711	-2.33127	0.47638	C	1.74838	0.02206	-0.75444
C	5.33011	-2.17474	0.23156	C	3.20064	-0.08749	-0.72053
C	7.68418	-1.27333	0.16649	C	1.08307	1.22455	-0.33292
C	7.10306	-0.13034	-0.36212	C	1.91529	2.23997	0.08966
C	5.71995	0.02140	-0.58945	C	3.33014	2.13289	0.12349
C	4.78418	-0.96675	-0.32370	C	4.01900	1.00242	-0.26344
C	3.36673	-0.77715	-0.59990	C	13.61158	-1.14213	0.82528
C	2.78653	0.27841	-1.27977	C	-3.71277	-0.49172	0.37253
C	1.38177	0.20747	-1.36740	C	-2.61534	0.51110	0.01685
C	0.84473	-0.90731	-0.75902	C	-1.24408	0.31737	0.00150
S	2.11473	-1.88703	-0.06109	C	-0.38843	1.37873	-0.35157
C	9.11486	-1.39027	0.39616	C	-0.95766	2.61892	-0.69829
C	9.86532	-2.49498	0.74469	C	-2.33407	2.81711	-0.68815
C	11.24947	-2.23462	0.86276	C	-3.16788	1.75907	-0.32515
C	11.59944	-0.92643	0.61334	C	-4.62500	1.66901	-0.21835
S	10.16908	0.01121	0.23329	C	-5.62611	2.61308	-0.44844
C	15.01455	2.63518	-0.78837	C	-6.95474	2.24709	-0.26099

C	16.46173	2.67213	-0.29750
C	17.46244	3.57777	-0.61668
C	18.74300	3.43977	-0.05815
C	18.98552	2.37221	0.81749
C	17.98814	1.45543	1.14544
C	16.72019	1.60744	0.58546
C	15.49581	0.81754	0.73281
C	15.22058	-0.32753	1.48230
C	13.94519	-0.87791	1.43943
C	12.92717	-0.31083	0.64719
C	13.22058	0.84520	-0.10449
C	14.48741	1.40065	-0.05697
C	14.24549	3.90741	-0.36985
C	14.94387	2.45466	-2.32045
N	-16.13225	-0.09157	0.17456
S	-15.82272	-1.66888	-0.13381
N	-14.18868	-1.60469	-0.21873
C	-13.82475	-0.33749	-0.01029
C	-14.96082	0.54428	0.22012
C	-12.47675	0.16991	0.00351
C	-12.37219	1.53241	0.23586
C	-13.47550	2.38514	0.45235
C	-14.79040	1.94947	0.46719
C	-15.89765	2.86339	0.72250
C	-15.81687	4.13682	1.24534
C	-17.06170	4.80973	1.32184
C	-18.11636	4.06661	0.86178
S	-17.57588	2.49503	0.33500

C	-7.31075	0.95045	0.15557
C	-6.28956	0.00580	0.37265
C	-4.96463	0.36616	0.18981
C	-3.71107	-1.69171	-0.60031
C	-3.57445	-0.98956	1.82750
N	-8.65441	-1.69139	-0.52017
S	-9.74621	-2.89806	-0.71735
N	-11.07777	-2.16654	-0.10150
C	-10.70468	-0.94145	0.28126
C	-9.29827	-0.66477	0.04357
C	-11.57345	0.03517	0.86182
C	-10.99327	1.23861	1.17003
C	-9.61439	1.51822	0.94051
C	-8.73452	0.61449	0.38550
C	-13.02157	-0.27883	1.10058
H	11.18190	-2.34058	1.10952
H	13.00407	1.39304	0.03687
H	10.78704	2.38859	-0.39551
H	8.21428	2.94458	-0.72513
H	5.75925	2.96829	-0.87039
H	5.58863	-1.12537	0.42690
H	8.50516	-2.31532	-1.32396
H	7.30303	-3.02693	-0.23608
H	9.01835	-3.40641	-0.02591
H	8.28745	-1.02197	2.81586
H	8.89336	-2.63956	2.42336
H	7.17744	-2.26524	2.21526
H	1.47466	3.16220	0.44929

C	-11.31563	-0.67851	-0.21610	H	3.88714	2.97937	0.50714
C	-11.24791	-1.98994	-0.64017	H	13.59938	-1.85190	1.65608
C	-9.93012	-2.48707	-0.75975	H	14.35507	-0.37303	1.04493
C	-8.95251	-1.57627	-0.42727	H	13.95505	-1.68650	-0.06149
S	-9.68912	-0.06276	0.05970	H	-0.82025	-0.64242	0.26541
C	-19.56438	4.44604	0.80306	H	-0.30935	3.43174	-1.00374
C	19.82929	4.43896	-0.37738	H	-2.74353	3.78123	-0.96869
H	-1.30009	0.72455	-0.81552	H	-5.38333	3.61726	-0.77821
H	-0.17055	-3.41627	-0.48944	H	-7.73574	2.97058	-0.46337
H	-2.53089	-4.07656	-0.33350	H	-6.54971	-0.99614	0.68750
H	-5.17285	-4.24692	-0.24114	H	-3.80638	-1.35708	-1.63565
H	-7.60302	-3.89084	-0.18759	H	-4.54383	-2.36495	-0.38032
H	-7.03887	0.34936	-0.67276	H	-2.78136	-2.25946	-0.50817
H	-4.10192	0.67018	1.42900	H	-3.57484	-0.15381	2.53102
H	-5.09596	1.77443	0.46431	H	-2.64192	-1.54573	1.95576
H	-3.33266	1.89103	0.40167	H	-4.40194	-1.65590	2.08554
H	-4.22980	0.26746	-2.89398	H	-11.60059	2.01783	1.61821
H	-3.40903	1.65287	-2.15534	H	-9.24294	2.49034	1.24268
H	-5.17214	1.53593	-2.09345	H	-13.53651	0.57097	1.55083
H	7.73705	0.70848	-0.62467	H	-13.13112	-1.14309	1.76244
H	5.39020	0.97030	-0.99234	H	-13.52583	-0.53514	0.16408
H	3.35946	1.07851	-1.72809				
H	0.78390	0.93883	-1.89469				
H	9.42082	-3.46609	0.89697				
H	11.97611	-3.00078	1.09807				
H	17.26686	4.39824	-1.30101				
H	19.97524	2.25770	1.24753				
H	18.20503	0.63741	1.82388				

H	15.98291	-0.78414	2.10386	
H	13.72375	-1.74882	2.04439	
H	12.45354	1.28309	-0.73441	
H	14.28211	4.05028	0.71248	
H	13.19675	3.84041	-0.67186	
H	14.67781	4.79094	-0.84718	
H	15.48150	1.55752	-2.63552	
H	15.38612	3.31581	-2.82859	
H	13.90516	2.36595	-2.65019	
H	-11.39055	1.99170	0.23716	
H	-13.26611	3.43765	0.59478	
H	-14.88828	4.57677	1.58346	
H	-17.17783	5.81232	1.71409	
H	-12.13101	-2.56622	-0.86787	
H	-9.70621	-3.48564	-1.11061	
H	-19.69500	5.46123	1.18230	
H	-20.18398	3.77726	1.40789	
H	-19.95136	4.41509	-0.21981	
H	19.78544	4.75491	-1.42270	
H	20.82121	4.02346	-0.18842	
H	19.72765	5.33989	0.23791	

In **Figure SI1**, the 1PA spectra of the copolymers in chloroform are shown, along with the oscillator strengths calculated at the TD-PCM-M06/6-311G++(d,p) level. A red shift of approximately 0.28 eV was observed in the theoretical transition energy compared to experimental 1PA. This behavior was expected, as it is well known that TD-DFT calculations tend to overestimate the transition energy. Notably, these calculations were performed better to understand the electronic band nature of the copolymers. The complete results, including transition energy, wavelength, and oscillator strength, are listed in **Table SI2**.

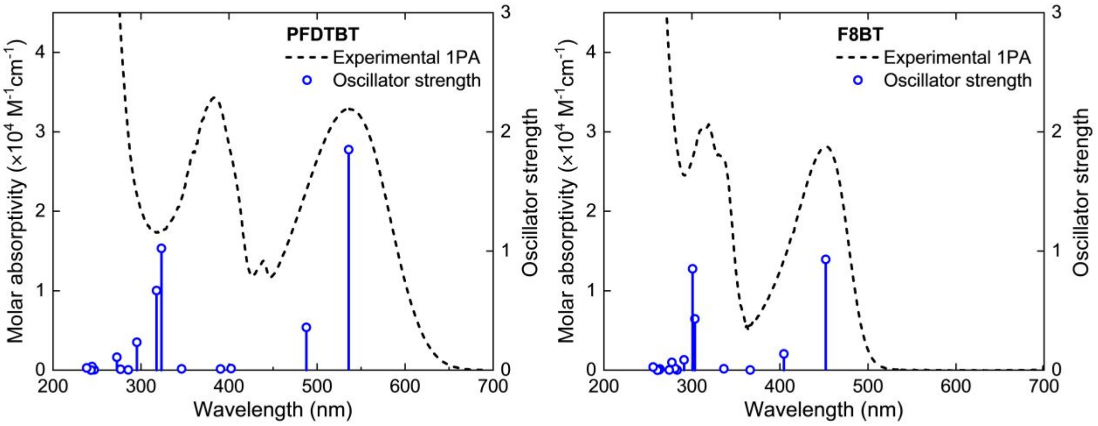

Fig. SI1 – One-photon absorption spectra (black dashed lines, left axis) of the copolymers in chloroform. The blue vertical lines with circles (right axis) represent oscillator strengths obtained from calculations at the TD-PCM-M06/6-311G++(d,p) level. The oscillator strength energies were blue-shifted by 0.35 eV for **PFDTBT** and 0.21 eV for **F8BT**.

Table SI2 - Theoretical photophysical properties of oligomer **PFDTBT** and **F8BT** obtained by TD-PCM-M06/6-311G++(d,p) calculation in chloroform medium, such as transition energy, transition wavelength, and oscillator strength.

Oligomer **PFDTBT**			Oligomer **F8BT**		
TD-PCM-M06/6-311G++(d,p) medium chloroform					
Energy (eV)	Wavelength (nm)	Oscillator Strength	Energy (eV)	Wavelength (nm)	Oscillator Strength
1.965	631	1.850	2.533	489	0.930
2.127	583	0.359	2.806	442	0.137
2.493	497	0.013	3.072	404	0.002
2.554	485	0.010	3.317	374	0.012
2.810	441	0.011	3.638	341	0.430
2.965	418	1.022	3.667	338	0.851
3.004	413	0.668	3.774	329	0.086
3.176	390	0.234	3.867	321	0.002
3.257	381	0.002	3.898	318	0.015
3.335	372	0.008	3.940	315	0.065
3.372	368	0.109	3.977	312	0.002
3.627	342	0.004	4.119	301	0.011
3.654	339	0.030	4.132	300	0.003
3.664	338	0.001	4.156	298	0.000

3.719	333	0.019	4.226	293	0.027

Figure SI2 presents two oligomeric structures of **PFDTBT** and **F8BT**: one containing three repeating units and the other containing two. As the chain length increases, the structure becomes progressively more twisted, losing its planarity. This effect is assumed to be even more pronounced in chains with more than ten repeating units.

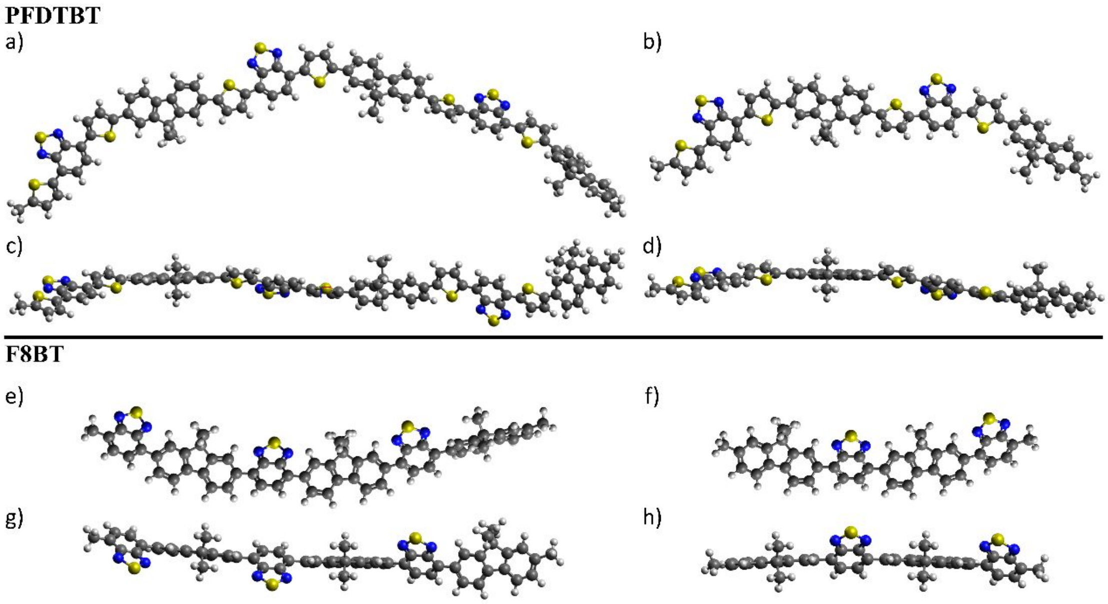

Fig. SI2 – Optimized molecular structures of oligomer **PFDTBT** and **F8BT** in chloroform. Atoms are represented by spheres of different colors: hydrogen (white), carbon (gray), nitrogen (blue), and sulfur (yellow). Where the labels are a), b), e), and f) frontal images, c), d), g), and h) images after a 90-degree rotation in the vertical direction.
